# DigEST: Digital plug‐n‐probe disease Endotyping Sensor Technology

**DOI:** 10.1002/btm2.10437

**Published:** 2022-11-05

**Authors:** Antra Ganguly, Tahmineh Ebrahimzadeh, Jessica Komarovsky, Philippe E. Zimmern, Nicole J. De Nisco, Shalini Prasad

**Affiliations:** ^1^ Department of Bioengineering University of Texas at Dallas Richardson Texas USA; ^2^ Department of Biological Sciences University of Texas at Dallas Richardson Texas USA; ^3^ Department of Urology University of Texas Southwestern Medical Center Dallas Texas USA

**Keywords:** Boolean logic, digital biosensor, disease endotyping, electrochemical impedance spectroscopy, random‐forest

## Abstract

In this work, we propose a novel diagnostic workflow—DigEST—that will enable stratification of disease states based on severity using multiplexed point of care (POC) biosensors. This work can boost the performance of current POC tests by enabling clear, digestible, and actionable diagnoses to the end user. The scheme can be applied to any disease model, which requires time‐critical disease stratification for personalized treatment. Here, urinary tract infection is explored as the proof‐of‐concept disease model and a four‐class classification of disease severity is discussed. Our method is superior to traditional enzyme‐linked immunosorbent assay (ELISA) as it is faster and can work with multiple disease biomarkers and categorize diseases by endotypes (or disease subtype) and severity. To map the nonlinear nature of biochemical pathways of complex diseases, the method utilizes an established supervised machine learning model for digital classification. This scheme can potentially boost the diagnostic power of current electrochemical biosensors for better precision therapy and improved patient outcomes.

## 1 INTRODUCTION

As observed in the recent COVID‐19 pandemic, point of care (POC) tests are changing the face of healthcare by making home‐based screening and routine health monitoring possible. The POC market is rapidly growing and is projected to reach 50.6 billion USD by 2025.^1^ According to a recent paper by Nguyen et al., the commercialization success of POC devices depends on three critical factors: (i) sample handling, (ii) biomarker detection, and (iii) signal reading.^2^ Most of the current POC biosensors lack in the third point, that is, the ease of reading the output signal. While many POC tests give an easy‐to‐read yes/no output, such as dipsticks for urinary tract infection (UTI), more powerful and accurate mathematical models for biomarker‐based disease diagnosis are being developed, and these tests are being replaced by technology with fully quantitative continuous‐valued (i.e., analog) outputs for biomarker concentrations. However, while analog signals can contain a high density of information, they are prone to noise and require larger memory storage. Drawing parallels from the successful digital transformation in the telecommunication industry over five decades ago, we propose a novel strategy—DigEST—to digitize the output of current electrochemical biosensors by discretizing the analog biomarker level information into digital states. This is attractive because a digital output corresponding to the disease endotype (which is a subtype of a health condition governed by a distinct pathobiological mechanism^3^) or severity state will allow for clear and actionable outcomes for the end user. Further, digitization will reduce the chance of misdiagnosis as digital signals are more immune to noise and require very large fluctuations for the output to jump from one state to another. By using the DigEST workflow, complex multibiomarker information can be “digested” and translated into a single clear, actionable outcome for the clinician and/or patient.

In recent years, there has been increased traction in the research for host response‐based diagnostics for personalized treatment and precision medicine.^4^ There has been a shift toward customized disease management through endotype‐driven diagnostics in which triage is initiated keeping in mind the dynamic biological variability due to genetic predisposition, treatment response, and triggered biological pathways.^5^, ^6^, ^7^ Disease endotypes are characterized by immunological biomarkers associated with the complex biological pathways at play.^6^, ^7^, ^8^ Leveraging this, we put forward a versatile, plug‐n‐probe modular workflow using traditional electrochemical biosensors to map the chronological spread of infection using urinary tract infection or UTI as the proof‐of‐concept infectious disease model. UTIs are among the most common bacterial infections and occur when certain bacterial species enter and multiply in the urinary tract.^9^ UTIs can be classified based on their location in the urinary tract as lower UTI (bladder and urethra) or upper UTI (kidneys or ureters).^9^, ^10^, ^11^ Lower UTIs are more common and can translate into upper UTIs as the causative pathogen ascends from the lower urinary tract to the kidneys resulting in pyelonephritis (kidney infection) ultimately spreading to the bloodstream causing urosepsis and even death.^12^, ^13^ The proposed device relies on tracking the combined fluctuations of the levels of three inflammatory urine biomarkers (PGE2, IL‐6, and CRP) to map the biological cascade events triggered upon infection (this is discussed further in the next section). UTI endotype changes as the pathogen travel from the lower to the upper urinary tract and has been classified as four digital states at the output viz., healthy, presymptomatic, symptomatic lower UTI, and pyelonephritis/systemic spread. An approximation of the biochemical cascade events associated with a disease endotype resulting in the release of key biomarkers was constructed using a discrete Boolean logic‐based model. This method is commonly used in systems biology and was preferred over other mathematical models built with continuous differential equations because such models require that numerous parameters are known beforehand and this information is largely unavailable for complex disease models affecting multiple biomarkers. We believe that DigEST can transform the fields of POC diagnostics and combinatorial disease biosensors by enabling disease endotyping and severity stratification for timely treatment, reduced costs, and hospital stays.

The current clinical workflow for the management of UTIs is based on the gold standard technique of culture‐based analysis of the presence of uropathogenic bacteria combined with reported symptoms. The goal of this work was to propose an alternative self‐monitoring workflow to address the gap in the current clinical UTI diagnosis and management workflow. At‐home routine self‐monitoring of UTI symptoms is particularly valuable for recurrent UTI (rUTI) patients. rUTI is characterized by ≥3 episodes of symptomatic UTIs in 12 months or ≥2 episodes of symptomatic UTIs in 6 months.^14^, ^15^ Through this work, we propose a rapid and novel UTI and rUTI management system which expedites clinical decision‐making and enables immediate diagnosis. By utilizing the proposed DigEST, rUTI patients can monitor their urine in the comfort of their own homes and get a preliminary screening result that will guide them in seeking care to ensure timely and appropriate prescription of antibiotics for increased treatment success rates.

**FIGURE 1:**
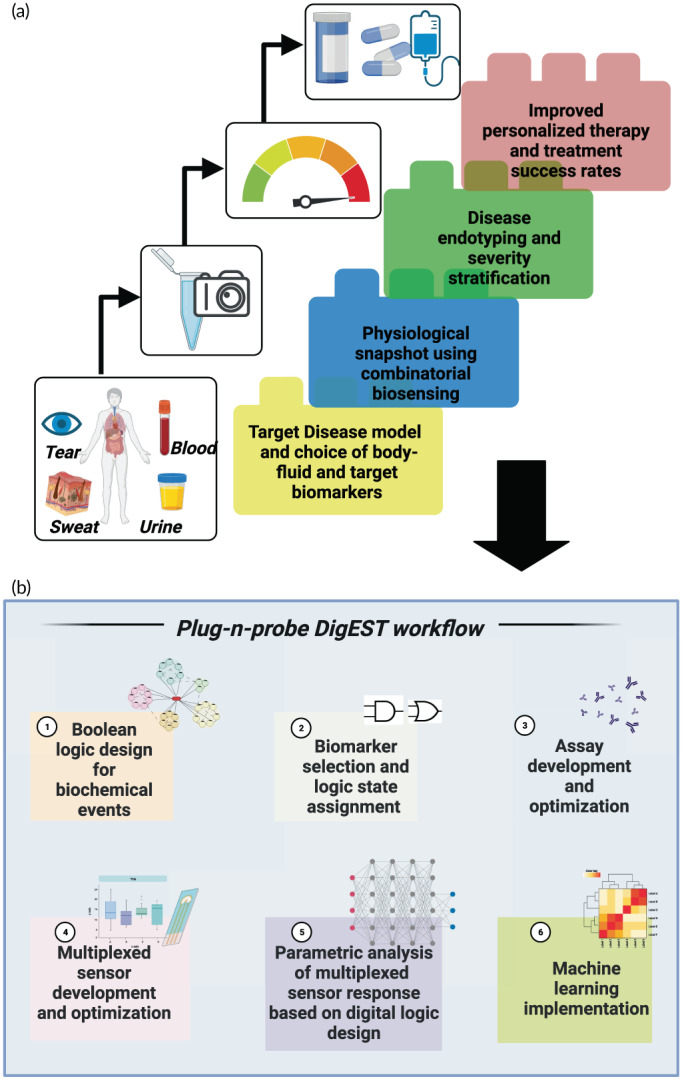
DigEST workflow. Schematic showing the generalized workflow for DigEST implementation to different disease models for precision and personalized therapeutics. Created using Biorender.com

DigEST operates on the principle of affinity‐based electrochemical biosensing^16^, ^17^ and allows for the detection of inflammatory biomarkers in the patient's urine in less than 5 min. The device requires no preprocessing or filtering of the urine sample or any other sample preparation by the end user. Figure 1 shows the operation of the proposed DigEST system for POC management of UTIs through multiplexed detection of key inflammatory biomarkers.

**FIGURE 2:**
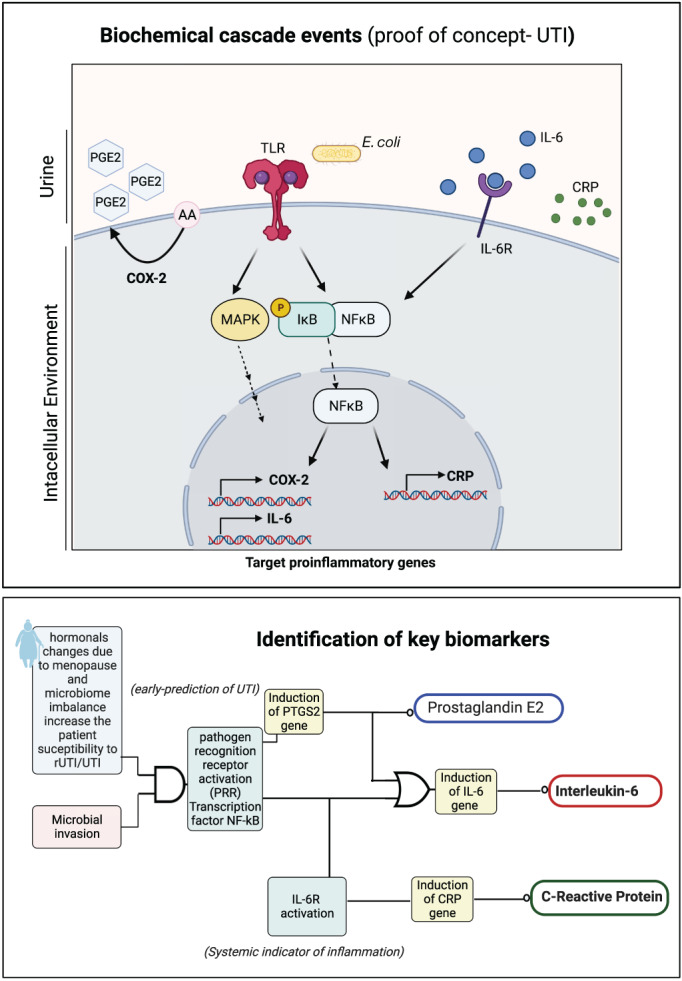
Concept diagrams for programming the circuit for Boolean logic and digital disease state classification. Partially created using Biorender.com. The events and biomarkers in the figure have been selected for demonstrating proof of concept (other MAPK include: ERKs, JNK, P38). These will change based on disease model of interest and levels of endotyping.

**FIGURE 3:**
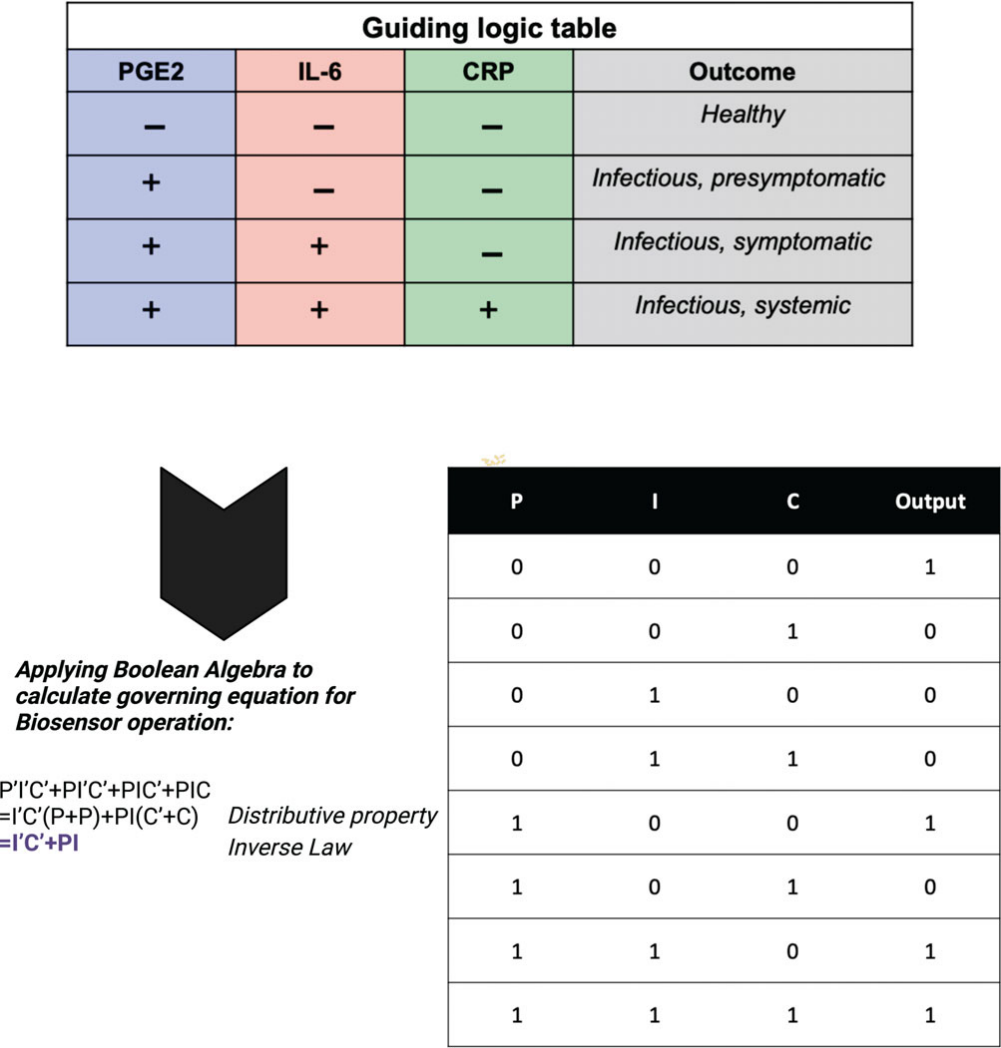
Boolean logic for programming USENSE for disease endotyping. Key biomarkers and associated pathways were identified based on which Boolean logic was developed for programming the biosensor system. Partially created using Biorender.com. The perceived outcomes in guiding logic table are representational and have been arbitrarily assigned. These states will change based on biomarkers and disease model of interest.

Three biomarkers prostaglandin E2 (PGE2),^12^, ^14^ interleukin‐6 (IL‐6),^18^, ^19^, ^20^, ^21^, ^22^ and C‐reactive protein (CRP)^23^, ^24^, ^25^, ^26^, ^27^ have been chosen for this study. These biomarkers are associated with the host immune response to UTI or rUTI. The choice of IL‐6, PGE2, and CRP was based on published data associating these biomarkers with UTI and the advanced understanding of the signaling pathways regulating their induction in response to infection (see Figure 2). DigEST is intended as a versatile plug‐n‐play UTI management system that can be used to measure additional UTI‐relevant urinary biomarkers as the biomarker discovery associated with UTI and rUTI evolves. Depending on the levels of the three biomarkers, the device outputs a disease state (1 = “healthy,” 2 = “infectious, asymptomatic or pre‐symptomatic,” 3 = “infectious, symptomatic” or 4 = “infectious, systemic”), which is associated with the predicted severity and spread of the UTI (see Figure 3). Depending on the predicted disease state, the patient along with their doctor can use this information to develop an effective treatment strategy. The proposed technology is superior to the alternative near‐patient, POC urine dipstick using nitrite or leukocyte esterase detection, which suffers from high false positive rates and results in unnecessary antibiotics use,^13^ for UTI diagnosis in two ways. First, the device quantifies the levels of three biomarkers and gives out a numerical readout, unlike the qualitative “yes” or “no” output of traditional dipsticks. Also, it integrates biomarker concentration information to output a disease state stratification that can easily be interpreted by the patient or clinician. Some of the key features of the proposed device include label‐free detection and easy handling, rapid response (<5 min), UTI severity prediction, multiplexed output, and versatile plug‐and‐play capability (see Section 3).

Figure 1 shows the generalized workflow for using DigEST for disease endotyping and severity stratification. The DigEST method is modular and versatile and has a plug‐n‐probe design that can be applied to a host of diseases and a variety of body fluids. Disease endotyping can be achieved through a series of six steps: (1) first, the active signaling and biochemical pathways have to be identified for the healthy state and the classes of unhealthy states corresponding to the target disease model. (2) Next, three key biomarkers involved in these biochemical events need to be identified as target analytes of interest for which individual biosensors can be developed. Based on the combination of biomarkers, severity and endotypes need to be defined by identifying associations between individual biomarkers and endotype. These endotypes or stratification states are then assigned digital logic states for example, states 1 through 4 corresponding to “healthy,” “asymptomatic or pre‐symptomatic,”, “symptomatic,” and “symptomatic, systemic,” respectively. (3) This is followed by the development and optimization of the assay elements. (4) Next, the electrochemical biosensor is developed and optimized to achieve target metrics such as the limit of detection and detection ranges for each biomarker such that the entire physiologically relevant range is covered. (5) Then, the sensor parameters are chosen which allow for differentiation between the output states with minimum interference. DigEST uses electrochemical impedance spectroscopy as the transduction mechanism as it affords additional interfacial parameters such as interfacial resistance and capacitance besides the real and imaginary parts of the complex impedance output. (6) Finally, the data collected is organized and labeled according to the target classes viz. the output disease states and fed to a supervised machine learning model for training, testing, and validation.

## 2 RESULTS

### 2.1 Identification and selection of key biomarkers for proof of concept

The choice of biomarker is critical for the successful implementation of the DigEST workflow. A diverse set of inflammatory molecules is expressed in response to UTI. For proof of concept, we have chosen PGE2,^12^, ^14^ IL‐6,^19^, ^20^, ^21^, ^25^ and CRP,^23^, ^24^, ^26^, ^27^, ^28^ which are established inflammatory biomarkers studied in the urine associated with UTI and its severity.

Figure 2 shows the relationship between the three biomarkers that we selected for UTI diagnosis. During UTI, lipopolysaccharides (LPS) present in the outer leaflet of the outer Gram‐negative bacterial membrane are detected by pathogen recognition receptors (PRR), such as the toll‐like receptor 4 (TLR4).^29^ TLR4 in turn activates myeloid differentiation primary response gene 88 (MyD88).^30^ MyD88 recruits tumor necrosis factor receptor‐associated factor 6 (TRAF6), which then leads to mitogen‐activated protein kinase (MAPK) and IκB kinase (IKK) complex activation.^30^ MAPK and IKK activation facilitate translocation of activator protein 1 (AP‐1) and nuclear factor‐κB (NF‐κB) to the nucleus, respectively.^31^ AP‐1 and NF‐κB promote the expression of inflammatory cytokine genes, IL‐1β, IL‐6, IL‐8, COX‐2, iNOS, TNF‐α, CRP, and IL‐10. Transcriptional induction of CRP expression may also occur in response to IL‐6/IL‐6R interaction.^32^, ^33^ PI3K–protein kinase B (PkB)/Akt pathway plays a critical role in IL‐6 signal transduction and NFκB activation.^22^ Cyclooxygenase 2 (COX‐2) enzyme catalyzes a key step in the conversion of arachidonic acid (AA) into prostaglandins, including prostaglandin E2 (PGE2).^34^

PGE2 along with other cytokines triggers inflammation in response to infection. PGE2 has been reported as a hallmark of acute inflammation during UTI.^12^ COX‐2 pathway activation is critical in determining the disease outcome and patient susceptibility to the recurrent UTI in murine models and human.^12^, ^14^ PGE2 acts via four different receptors (EP1‐4) and triggers a series of proinflammatory responses for acute inflammation. IL‐6 is a multifunctional cytokine with broad‐ranging effects, which has a central role in the initiation phase of infection. IL‐6 is known as an early marker of tissue injury in many infections like COVID‐19.^35^ IL‐6 is an established inflammatory biomarker^19^, ^21^, ^22^, ^25^, ^36^, ^37^, ^38^, ^39^ expressed in urine and was found to be a promising biomarker to detect the transition from asymptomatic bacteriuria to symptomatic urinary tract infection in older adults.^40^ C‐reactive protein (CRP) is an acute phase protein that is a marker of systemic inflammation in the patient's body.^41^ CRP has been studied as an inflammatory biomarker in the literature^23^, ^24^, ^25^, ^26^, ^28^ and has been shown to differentiate between lower and upper UTI infections.^28^

### 2.2 Building the assay for multiplexed detection

At its core, the proposed UTI diagnostic system is an affinity‐based electrochemical biosensor. The biosensor is comprised of a standard screen‐printed three‐electrode system with gold working and counter electrodes and silver reference electrodes (Metrohm 220 AT). Highly specific monoclonal antibodies were chosen as capture probes for affinity capture of the target antigens (PGE2, IL‐6, and CRP) expressed in test urine samples. The antibody–antigen binding induces a subtle modulation of the electrode–urine buffer interface as a function of the concentration of the target antigen present in the urine sample.

The transduction of the antibody–antigen binding event is achieved by monitoring the interfacial modulation of the electrode–urine interface as a function of the binding. A standard three‐electrode electrochemical sensor has been used for the technological proof of concept and the established technique of electrochemical impedance spectroscopy (EIS) has been used. This is transduced by the powerful electroanalytical technique of EIS, which detects and correlates the subtle changes in the interfacial behavior to the levels of the analyte of interest (PGE2, IL‐6, CRP for this study). To bind the monoclonal antibody capture probe to the measuring electrodes, a self‐assembled monolayer of a homobiofunctional and amine‐reactive thiol crosslinker, DSP (dithiobis(succinimidyl propionate)) was developed on the working electrode.^42^, ^43^

**FIGURE 4:**
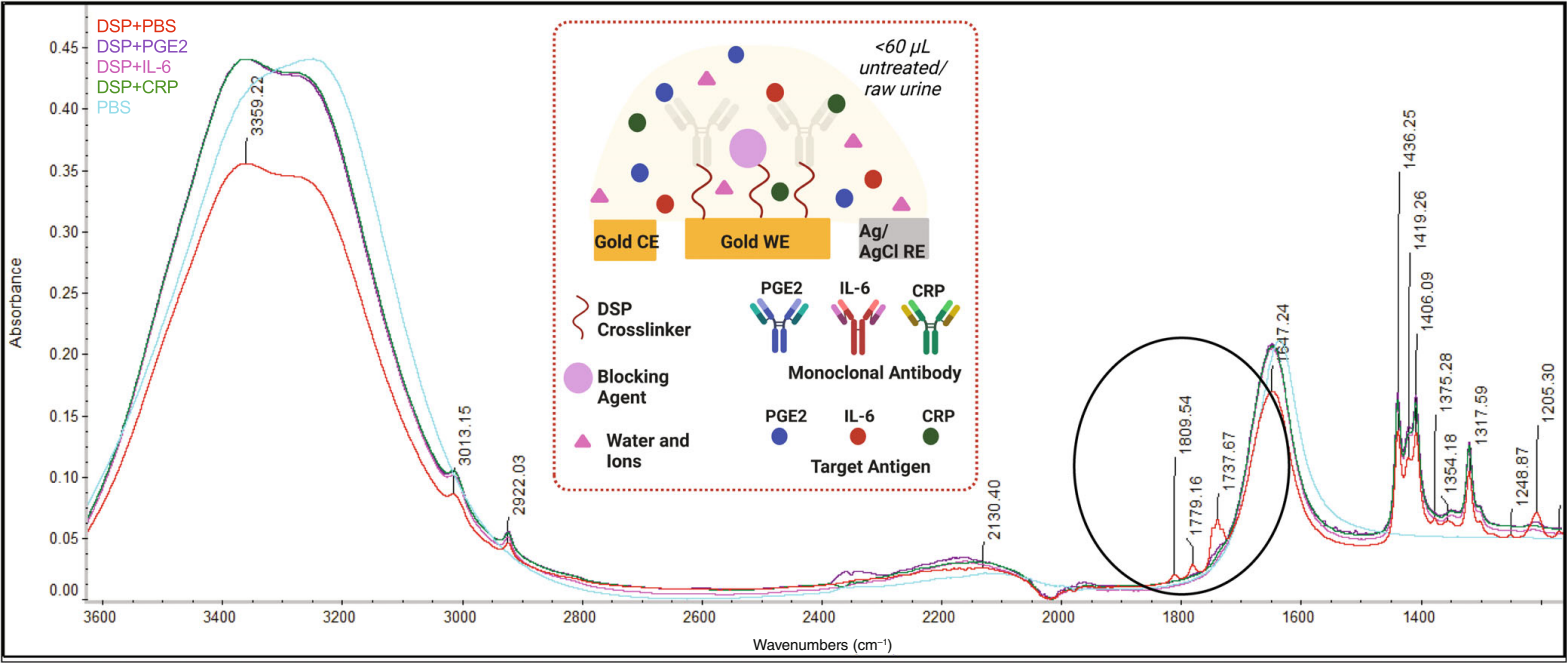
DigEST assay stack and validation of binding chemistry. Schematic of the electrochemical urinary tract infection (UTI) biosensor system and attenuated total reflectance Fourier transform infrared (ATR‐FTIR) spectra of the crosslinker and target analytes. Partially created using Biorender.com

To ensure successful immobilization and binding of the monoclonal antibodies with the DSP crosslinker on the gold working electrode, attenuated total reflectance Fourier transform infrared spectroscopy (ATR‐FTIR) was used. Through this technique, the infrared spectra of monoclonal antibody (mAb) and the DSP crosslinker were obtained and the characteristics peaks were identified to validate the binding chemistry. Liquid samples of DSP crosslinker, DSP bound to PGE2 mAb, IL‐6 mAb, and CRP mAb were studied. PBS was studied as the negative control. Figure 4 shows the schematic representation of the elements of the immunosensor assay stack and the FTIR spectra of all the combinations DSP‐mAb conjugates, DSP crosslinker, and PBS control overlayed on each other. All significant peaks used to validate the successful bonding of the crosslinker and mAb capture probes have been annotated. The spectra of DSP‐mAb conjugates of PGE2, IL‐6, and CRP in PBS respectively compared to the spectra of DSP (mixed in PBS) have been discussed in the supplementary information S1. In this way, the successful immobilization of the monoclonal antibodies to the crosslinker was validated before proceeding with the electrochemical studies.

### 2.3 Electrochemical characterization and calibration of sensor response using EIS

Non‐Faradaic EIS was used to transduce the biochemical activity of antigen–antibody binding at the electrical double layer interface. This was done to ensure label‐free operation to ensure that no sample preparation is required for the end user since non‐Faradaic mode eliminates the need for redox tagging to get a measurable signal. Further, EIS was chosen among other electroanalytical techniques because of two main reasons. First, EIS is a highly established powerful AC‐based technique to detect subtle binding interactions at the electrical double layer interface and has been reported for sub picomolar biomarker concentration quantification^44^ and has been used widely for biosensing using a variety of capture probes including antibodies, aptamers, and so on.^45^ Second, as a proof of concept, we have utilized a standard, widely used screen‐printed electrodes based three‐electrode electrochemical cell (Metrohm 220 AT) for building our plug and play diagnostic system without any sensor modification for signal enhancement to make it simple, versatile for easy, universal use. Since the response of the unmodified sensor is very subtle (in ohms), EIS was chosen to measure small changes in response corresponding to the immunological biomarker levels expressed in human urine. Thus, the output response for all three biomarkers was studied as the modulus of impedance. EIS studies were done for a wide frequency range of 1 MHz to 1 Hz. The 100 Hz was used as the optimal frequency for sensor calibration.

**FIGURE 5:**
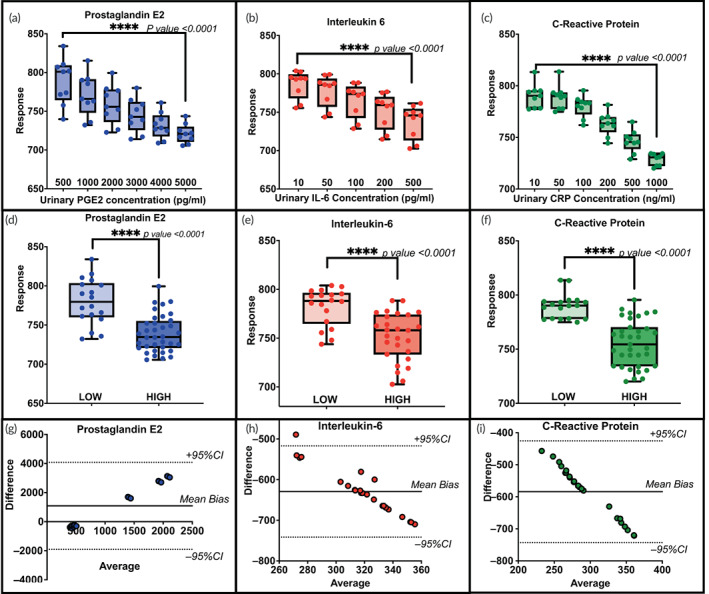
Detection of urinary PGE2, IL‐6, and CRP. (a–c) Calibrated dose–response curves using electrochemical impedance spectroscopy (EIS); (d–f) *t*‐test analysis for low versus high inflammation and (g–i) Bland Altman analysis comparing the response from EIS and enzyme linked immunosorbent assay (ELISA) for all three biomarkers

Figure 5a–c shows the calibrated dose response corresponding to PGE2, IL‐6, and CRP levels expressed in pooled human urine samples obtained from >3 healthy human donors. The urine samples were prepared by spiking the urine samples with physiologically relevant levels of the three biomarkers. PGE2 was spiked in the range of 500–5000 pg/ml. IL‐6 was spiked in the range of 10–500 pg/ml. CRP was spiked in the range of 10–1000 ng/ml. Raw or unspiked pooled human urine sample was used for the baseline dose (also known as zero doses) in the calibration studies. From Figure 4a–c, a dose‐dependent decrease in the sensor response (i.e., the modulus of impedance at 100 Hz) was obtained for all three biomarkers. One‐way ANOVA analysis showed a significant difference between the doses with a *p* value < 0.0001 for PGE2, IL‐6, and CRP, respectively. Pairwise *t*‐tests showed a significant difference (*p* < 0.0001) between different doses for each biomarker. Figure 4d–f shows the *t*‐test comparison of low and high levels of inflammation corresponding to low and high concentrations of the biomarkers expressed in human urine. Clearly, the sensor can differentiate between low and high levels of inflammation and infection. Thus, it was concluded that the sensor can quantify a wide dynamic range of target biomarkers of interest inflammation using <60 μl of urine in less than 5 min without any labeling or additional sample preparation.

### 2.4 Human subject studies and comparison with ELISA

After calibrating the sensor using spiked samples, human subject samples from 10 post‐menopausal women with a history of UTI or rUTI were tested. The same samples were also studied using ELISA, an established laboratory technique of biomarker level quantification in human samples. The response from EIS was compared with that from ELISA using Bland Altman (BA) analysis. Figure 4g–i shows the BA analysis represented as the difference versus the average between the two methods. Almost all the data points corresponding to all the samples (three replicates each) fell within the 95% confidence interval level control limits indicating high agreement between the two methods.

### 2.5 Testing performance of sensor for Boolean‐based endotyping studies

The biochemical cascade events triggered upon urinary tract infection have been discussed in Figure 4a. This has been used to identify key biomarkers as shown in Figure 4b. A proof‐of‐concept Boolean logic using universal AND and OR gates has been developed corresponding to the biological events shown in Figure 5a.

From Figure 4c, the truth table in Figure 3d has been developed. Upon solving using Boolean algebra, the corresponding Boolean equation has been obtained (P, I, and C represent the state of PGE2, IL‐6, and CRP respectively, and “‘” [left single quote] represents complement or NOT operation). This can be used to program digital logic at the output. As mentioned before, this can be extended to developing simple diagnostics for a gamut of disease models.

### 2.6 Comparison of sensor performance with EIS and ELISA for Boolean samples

The cocktail solutions corresponding to the four output digital states (LLL, HLL, HHL, and HHH) were tested using EIS and ELISA methods. Figure 5a,b shows the 3D scatter plot of the normalized response of ELISA and EIS with PGE2, IL‐6, and CRP levels expressed along the X, Y, and Z axes, respectively. Each dot on the scatter plot represents the average response (obtained from *n* = 3 sensors with three intrasensor replicates each) for each of the four Boolean states. For example, consider the case representing the endotype corresponding to digital state 2 or “HLL.” “HLL” means that the test sample contains human urine with high PGE2, low IL‐6, and low CRP. As discussed earlier, this corresponds to the “infectious, asymptomatic” case. Thus, three X, Y, and Z axes have been discussed to represent the normalized values obtained for the sensor/ELISA kit corresponding to each biomarker.

**FIGURE 6:**
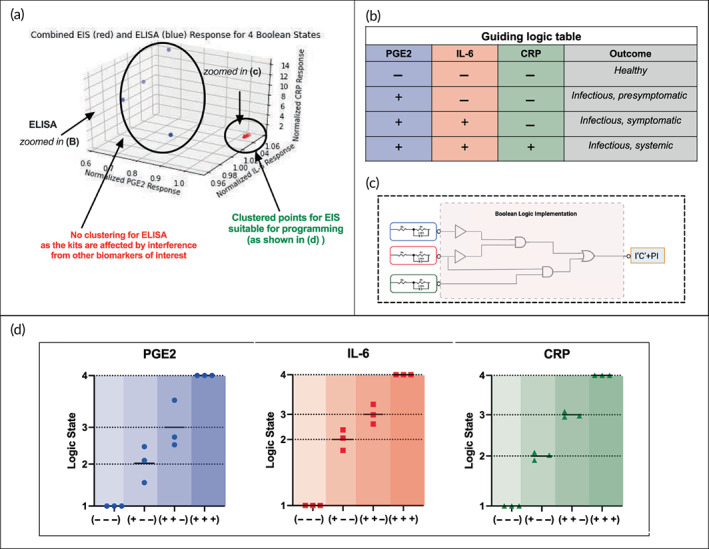
Boolean logic implementation. (a) Combined EIS (red) and ELISA (blue) response for four Boolean states. 3D scatter plots showing the response from EIS and ELISA for three different biomarkers depicted across the three axes. X, Y, and Z axes correspond to the response (i.e., Z_mod_ at 100 Hz for EIS and absorbance at 450 nm for ELISA) normalized to the baseline dose (unspiked pooled human urine sample) for PGE2 (X axis), IL‐6 (Y axis) and CRP (Z axis). (b) Truth table guiding programmable digital logic, (c) electronic circuit design for digital logic, and (d) 2D representation of the EIS response corresponding to each target biomarker normalized to the four output states of Boolean logic

Figure 6c. shows the inset with Figure 5a,b overlayed and zoomed in. As highlighted in the figure, the data points showing the response from EIS (red) are well clustered and are suitable for further programming. On the other hand, the response from ELISA showed no clustering. This is probably because three different kits were required to obtain the results for three different biomarkers and their results are affected by the interference from other biomarkers of interest.

The results from the EIS response shown in Figure 6b have been represented as three different plots corresponding to each biomarker. The EIS data for each biomarker have been normalized to the data for the healthy state, which is the first state or “LLL.” From the figure, it is clear that for all the biomarkers, EIS was capable of separating the four states and hence can be used for UTI severity thresholding and disease endotyping. The truth table corresponding to the levels of a given biomarker corresponding to each state has been indicated in the truth table in Figure 6e for reference. Figure 6e shows the digital implementation of the Boolean equation obtained for the proof‐of‐concept disease model in this study discussed in the previous section (calculated in Figure 5d), that is, *I′C′* + PI. Figure 6e also shows the electronic circuit design that can be used to implement the logic in a future POC UTI management device.

### 2.7 Characterization of the interfacial behavior for different digital states

Equivalent circuit fitting was done to obtain the transfer function of the electrochemical process in terms of electrical circuit elements, that is, resistance and capacitance (constant phase element to model a leaky capacitor). The interfacial modulation due to Ab‐Ag binding was found to show typical Randle circuit behavior with the solution resistance (R_s_) in series with the parallel combination of double layer capacitance (C_dl_ modeled as a constant phase element) and the leak resistance (R_p_). The circuit has been discussed in detail in the supplementary information S1. Zview software was used to fit the Randle circuit to obtain the fit parameters.

Figure S1 shows the variation in the circuit parameters R_s_, C_dl_, and R_p_ as a function of the four digital states viz., LLL (State 1), HLL (State 2), HHL (State 3), and HHH (State 4) for (a) PGE2, (b) IL‐6, and (c) CRP. For each of the subfigures, the color gradient (light or dark) reflects the variation in the levels (high or low) of the corresponding biomarker expressed in urine. For example, consider 6 (b) which shows the graph for IL‐6. Here, for States 1 and 2, the level of IL‐6 spiked in the cocktail is low, that is (L**L**L and H**L**L) and hence has been depicted in light red color. On the other hand, for States 3 and 4, the level of IL‐6 spiked in the cocktail is high, that is (H**H**L and H**H**H) and hence has been depicted in dark red color.

This equivalent circuit model analysis was done to evaluate whether the individual circuit elements could be tuned to calibrate the sensor for disease endotyping. The relation between Zmod and the circuit elements^45^ has been discussed in the supplementary information. From Figure S1a–c, it is evident that the solution resistance R_s_ (depicted by squares) is almost constant across the four digital output states. This is favorable as it indicates that EIS is truly mapping the interface and the output impedance is unaffected by the effect of the nonspecific molecules and ions in the bulk solution. Next, it was found that for 6(a) and 6(b), C_dl_ (triangle symbol) and R_p_ (circle symbol) were able to follow the low‐ and high‐dose concentrations for PGE2 and IL‐6 across the four different states. This is because EIS output is very specific to the affinity binding of the target antigen to the specific monoclonal antibody at the double layer interface. However, in Figure S1c, this effect is not observed, and the individual parameters are unsuitable for disease state endotyping.

### 2.8 Implementation of the sensor using supervised machine learning platform

From the results of the previous section, it was realized that simple circuit fitting was insufficient to model the complex events at the electrical interface corresponding to truly map the nonlinear behavior of the biological pathways for a given disease model. To solve this, the impedance data were fed and trained using a simple machine learning model. To avoid high computation costs, a simple random forest (RF) model was used for training. RF is an ensemble method that relies on multiple decision trees with varying numbers and depths to achieve the mapping of relationships between the inputs and the outputs. RF models are often used in real‐world research problems as they are highly immune to the effects of biases and variances because they give out a classification based on bootstrapping the outputs of multiple decision trees, instead of relying on a single tree.^46^

**FIGURE 7:**
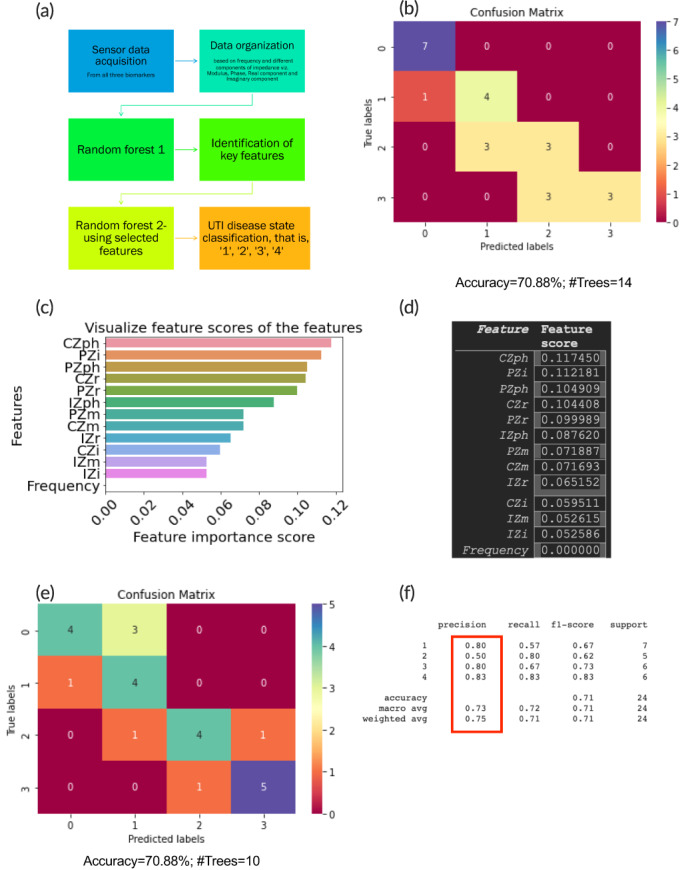
Machine learning analysis. Design and implementation of the machine learning model for UTI disease state endotyping

Figure 7a shows the flow chart for the machine learning model. The sensor data acquired from the three biomarkers corresponding to the four Boolean states were labeled as “1,” “2,” “3,” or “4” corresponding to the output digital state. Instead of using the individual circuit fit parameters discussed in the previous section, the real, imaginary, modulus, and phase values of impedance obtained for each target analyte were studied along with the frequency of operation. For proof of concept, two frequencies 10 and 100 Hz were studied to check if the frequency of operation in the low region affected the output digital state. These 13 features were studied as discussed in Table 1.

**TABLE 1:** Features studied using random‐forest model

Feature #	Feature name	Description
1	PZm	Zmod or modulus of impedance for PGE2
2	PZph	Zphase or phase angle (in degrees) of impedance for PGE2
3	PZi	Zimag or imaginary component of impedance for PGE2
4	IZr	Zreal or real component of impedance for IL‐6
5	IZm	Zmod or modulus of impedance for IL‐6
6	IZph	Zphase or phase angle (in degrees) of impedance for IL‐6
7	IZi	Zimag or imaginary component of impedance for IL‐6
8	IZr	Zreal or real component of impedance for IL‐6
9	CZm	Zmod or modulus of impedance for CRP
10	CZph	Zphase or phase angle (in degrees) of impedance for CRP
11	CZi	Zimag or imaginary component of impedance for CRP
12	Zr	Zreal or real component of impedance for CRP
13	Frequency	Frequency of operation, that is, 10 Hz or 100 Hz

The total size of the dataset was 120 rows and 13 columns. Data augmentation (a common technique used in machine learning data analysis to increase the amount of data by reorganizing already existing data for improved analysis without additional data collection) was done by subdividing the modulus of impedance used in sensor calibration into the real and imaginary and phase components to obtain a wide dataset for a more comprehensive analysis of the individual features. The RF model was tuned to obtain the maximum number of trees to achieve maximum accuracy for the given dataset. It was found that for the given dataset, 14 trees obtained the highest accuracy of 70.88% using 80% of the dataset for training and 20% of the dataset for testing. The confusion matrix in Figure 7b shows the output from a single RF. Since most of the samples fell in the main diagonal, it can be concluded that the model was capable of correctly predicting and classifying the output disease states with high accuracy.

Next, the model was used to analyze the importance of the different features for UTI endotyping. Figure 7c shows the bar graphs of the features in descending order of importance and Figure 7d shows the values of the feature scores corresponding to each feature under analysis. It was found that the frequency was the least important feature while the phase angle of CRP was the most important feature for our dataset.

After the identification of the key features, a new RF model (Random Forest 2) was developed wherein only the 10 most important features were studied and the relatively less important features were dropped. This was done to reduce the computation time and costs of analysis, which is especially important when dealing with larger datasets with multiple levels of classification. As depicted in Figure 7a, for RF‐2, 80% of the dataset was used for training while 20% was used for testing.

For the reduced and optimized RF, that is, RF‐2, fewer trees, that is, a total of 10 trees were required to reach the same maximum accuracy of 70.88%, keeping the tree depth constant (Figure 7e). By comparing the confusion matrices in Figure 7b,e, it can be clearly seen that more elements fall on the main diagonal for RF‐2 than that for RF‐1. This means that by cutting out the clutter by way of removing the unimportant features, the model was able to classify better all four digital states at the output. It is important to mention that all the classes were balanced in the input dataset. To study the metrics corresponding to the ability of the model to classify the four digital output states, “precision,” “recall,” and F‐1 scores were studied. The formula for each of these metrics has been listed as follows:

(1) $$\text{Precision}=\frac{\text{True positives}}{\text{True positives}+\text{False positives}}$$

(2) $$\text{Recall}/\text{Sensitivity}=\frac{\text{True positives}}{\text{True positives}+\text{False negatives}}$$

(3) $$F-1\text{Score}=2*\frac{\text{Precision}*\text{Recall}}{\text{Precision}+\text{Recall}}$$

The values of the metrics have been listed in Figure 7f for all four classes. A high precision value of > = 80% was found for States 1, 3, and 4, whereas it was 50% for State 2. In terms of recall, State 2 was higher than States 1 and 3 and lower than State 4. This may mean that for the given dataset, for State 2, false negatives were low; however, false positives were high. State 2 corresponds to the UTI endotype of “infectious, pre‐symptomatic” and is associated with biomarker levels corresponding to low inflammation. In this case, for home‐based use, it is more significant to ensure low false negatives such that early‐stage UTIs do not go undetected. For State 4, corresponding to the state of “infectious, systemic,” the values of precision, recall, and F‐1 score was the highest. This is desirable as this is the state corresponding to peak illness and has the causative microorganism spread systemically as reflected by high levels of all target inflammatory biomarkers in urine. In this way, the implementation of target disease stratification and endotyping was demonstrated using a simple statistical machine learning algorithm. To our knowledge, this is the first demonstration of direct integration of raw impedance values from an electrochemical biosensor to train a machine learning model for multistate disease classification. This model is versatile and can be extended to more complicated disease states with more stratification levels by studying a broader panel of biomarkers.

## 3 DISCUSSION AND CONCLUSION

In the current clinical workflow of management of UTIs that requires both the reporting of symptoms and detection of bacteria by the gold standard technique of urine culture, the patient, due to the lack of reliable self‐monitoring alternatives, must call to book an appointment with a clinician at the when feeling symptoms of a UTI. In the best‐case scenario (shown in Figure 7a), the patient visits the hospital or a doctor's office on the same day or the next day.

During this time, the UTI‐causing pathogen may ascend the urinary tract. At the hospital, the patient's urine is sent to a centralized lab for urine culture tests. The patient must wait for 2–4 days to get the results and is often prescribed a broad spectrum of antibiotics in the meantime. These prescriptions are often unnecessary, and the resulting overuse or misuse of antibiotics may result in antibiotic resistance and allergy.^47^ This often makes subsequent treatment more complicated, incurs unnecessary medical expenses, and reduces the chances of treatment success. In many unfortunate cases, the ineffectiveness of prescribed antibiotics results in longer hospital stays especially not suitable for high‐risk groups (such as pregnant, pediatric, and geriatric populations who are also highly vulnerable to UTIs) in the current pandemic situation. Delays in diagnosis can result in severe sequelae as the causative pathogen can travel from the lower urinary tract to the upper urinary tract to cause pyelonephritis and may ultimately spread to the bloodstream causing sepsis and even death.

**FIGURE 8:**
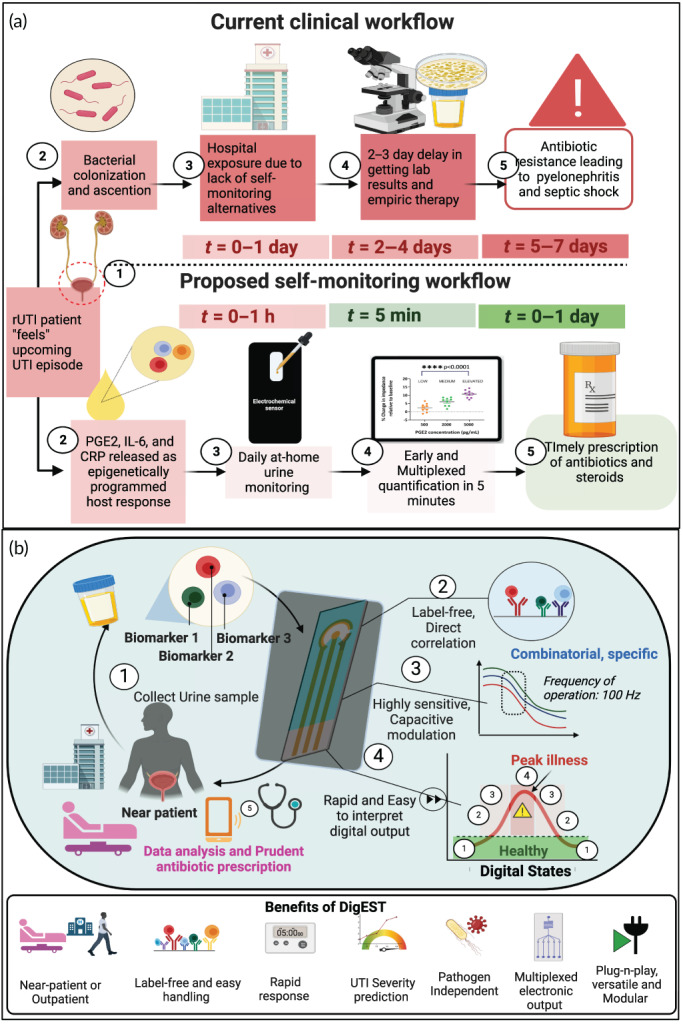
DigEST implementation, operation, and benefits. Schematic showing the (a) comparison between the current and the proposed clinical workflow for UTI diagnosis using DigEST and (b) Operation and key benefits of DigEST. Created using Biorender.com

In their recent work, Zhang et al. propose a rapid method of detecting UTIs for prompt initiation of therapy and improved antibiotic stewardship.^48^ However, their detection scheme relies on the visualization and analysis of *E. coli* in the urine sample. Since the method is phenotype‐driven, it lacks the ability to stratify UTI severity, which is critical for timely triage and treatment success. Also, this method only detects the presence of possible UTI‐causing bacteria in the urine but cannot detect if there is an immune response. The gold standard for UTI diagnosis requires the presence of a microbial pathogen in the urine and reported symptoms (i.e., dysuria, urgency, frequency), which are a result of the host immune response to infection. Bacteriuria in the absence of symptoms is called asymptomatic bacteriuria (ASB) and is not recommended to be treated with antibiotics. Thus, without accurate symptom reporting, which often occurs in elderly individuals, methods that only determine the presence of bacteria in the urine may misdiagnose ASB as UTI. Our work demonstrates a rapid, cost‐effective, disposable nonculture‐based electrochemical method to diagnose and manage UTI and recurrent UTIs by evaluating a panel of urine biomarkers (PGE2, IL‐6, and CRP) to map the severity of disease progression and disease endotyping (Figure 8).

This work demonstrates the development of a novel nonculture‐based method to diagnose and manage UTIs and rUTI by evaluating a panel of urine biomarkers to map the severity of disease progression and disease endotyping. The biosensing concept is versatile and modular and can be applied to any disease model, which requires time‐critical stratification for efficacious treatment. To demonstrate proof of concept, we have chosen UTI as the disease model of interest. UTIs are common infections that affect individuals of all age groups and are associated with frequent episodes of recurrence and relapse, especially in post‐menopausal women. UTIs, though a common cause of mortality worldwide, is often undiagnosed (especially in resource‐challenged settings due to limited access to laboratory culture tests) and when left untreated can result in severe sequelae such as sepsis and even death, as the causative pathogen ascends from the lower urinary tract to the upper urinary tract and ultimately spread into the bloodstream.^12^, ^13^, ^43^, ^49^, ^50^ Thus, tracking the progression of the disease, in a time‐critical fashion, is key for treatment success. To achieve this, we have developed a rapid UTI biosensor that measures inflammatory biomarkers which are expressed in urine in response to the underlying infection, regardless of the type of pathogen (commonly bacteria). Notwithstanding that there are several biomarkers associated with UTI, we have focused on three established inflammatory urinary biomarkers for our proof‐of‐concept study viz., PGE2, IL‐6, and CRP.^14^, ^25^, ^43^

The novelty of our sensor platform is that it stratifies UTI progression and gives out a digital state output corresponding to the disease endotype. This is a first‐of‐a‐kind biosensor that endotypes and classifies disease states based on severity considering levels of host inflammatory biomarkers.

Being nonculture based, it is independent of the causative pathogen. Further, The DigEST platform is scalable and future versions may employ alternate or additional biomarkers like LPS to get an even more accurate assessment of the disease state and might help in predicting the risk of UTI relapses. The biosensing concept is versatile and modular and can be applied to any disease model, which requires time‐critical stratification for efficacious treatment.

Non‐Faradaic, label‐free EIS was used to study the subtle interfacial modulation due to the specific affinity capture of the target analytes PGE2, IL‐6, and CRP by their corresponding highly specific monoclonal antibody. Four disease states were studied and arbitrarily assigned outcomes viz. State 1 through 4 corresponding to “healthy,” “infectious, pre‐symptomatic,” “infectious, symptomatic” and “infectious, systemic.” These states were chosen arbitrarily, and the proposed model can be extended to more biomarkers and varied classification states, as the field of UTI biomarker discovery evolves. Further, since the sensor system is an essential “plug‐and‐probe”, different analytes can be detected using different capture probes such as aptamers, molecular imprinted polymers, and so on.^17^

The sensor can be extended for endotyping other complex and heterogeneous diseases in other body fluid samples such as chronic obstructive pulmonary disease (COPD) in exhaled breath condensate patient samples. The sensitivity of the system can be enhanced for detecting trace molecules in femtomolar concentrations, if required, by replacing the generic sensor electrodes with decorated electrodes with surface modification to enhance surface reactivity and effective sensing surface area.^51^ For scaling the sensor performance, larger and more diverse patient cohorts need to be studied. For truly personalized disease management, key disease‐relevant biomarkers need to be identified and corresponding biological pathways need to be studied closely, as the biomarker discovery field evolves. This sensor demonstrates the use of random forest as the algorithm for machine learning‐guided classification of the output disease states. However, given the nature of the signal extracted from the electrochemical sensor, and the complexity of the events at the double layer interface, the dataset can be further augmented, and other traditional and deep learning statistical models can be employed to best optimize the tradeoff between application and the computational costs post‐implementation as POC device.

## 4 MATERIALS AND METHODS

### 4.1 Reagents and materials

Monoclonal α‐PGE2 antibody was purchased from Arbor Assays (Ann Arbor, MI, USA). Monoclonal antibodies for IL‐6 and CRP and their corresponding antigens were obtained from Abcam (Cambridge, MA, USA). The antibody was aliquoted and stored at 4°C until further use. HPLC purified PGE2 antigen was obtained in synthetic powder form from Sigma Aldrich (St. Louis, MO, USA). The crosslinker DSP (dithiobis (succinimidyl propionate)) and Phosphate Buffer Saline (PBS) were obtained from Thermofisher Scientific Inc. (Waltham, MA, USA). Artificial urine was prepared using the recipe (MP‐AU or multipurpose‐artificial urine) by Sarigul et al.^52^ and all the dilutions were prepared in deionized water. The urine buffer pH was adjusted using sodium hydroxide (10% NaOH) and Hydrochloric acid (10% HCl) obtained from Sigma Aldrich (St. Louis, MO, USA). Pooled human urine samples (pH ~6.5) were obtained from Lee Biosolutions (St. Louis). The sensor system was constructed using Metrohm 220 AT three‐electrode (gold working and counter electrode and silver reference electrode) screen‐printed sensor (Metrohm USA).

### 4.2 Experimental design of validation of binding chemistry

Nicolet 6700 FTIR (Thermo Fisher Scientific) was used for the ATR‐FTIR studies. A liquid sample of 10 mM DSP crosslinker dissolved in DMSO was used as the baseline for the analysis. A 1:1 mixture of the monoclonal antibody (corresponding to the target analyte u.e., PGE2, IL‐6, or CRP) in PBS and DSP crosslinker dissolved in DMSO was incubated for 2 hours. For both the baseline DSP spectrum and all the DSP‐mAb conjugate spectra, 256 scans were collected, for a wavelength range of 4000–600 cm^−1^, at a resolution of 4 cm^−1^.

### 4.3 Experimental design and optimization of EIS studies

The sensor consists of a standard three‐electrode system made up of gold working and counter electrodes and silver reference electrodes screen printed on a ceramic substrate. The 5 μl of 10 mM of DSP crosslinker (dissolved in DMSO) was incubated only on the working electrode for 2 h in dark for strong thiol bond formation. After DMSO wash and one 1X PBS wash, the monoclonal PGE2 antibody was immobilized on the gold working electrodes in 5 μl volume for overnight incubation for EDC‐NHS bond formation. The crosslinker concentration was selected based on the literature. ATR‐FTIR‐based spectroscopic validation (described in previous sections) was used to validate the suitability of the selected incubation time and antibody concentration for urine prostaglandin E2 biosensing. The volume of 5 μl for crosslinker and antibody was chosen to ensure that the entire working electrode (only WE) gets covered to ensure specificity of the response.

### 4.4 Experimental design and testing of Boolean studies profiles: Simulated Boolean profiles

To simulate the samples for different endotypes, four different cocktails of the urine samples were prepared by 1:1:1 mixing samples with varying levels of the three biomarkers between two states, that is, high (h) or low (L). Mathematically, for three biomarkers and two states of levels/inflammation, 2^3^ = 8 combinations are possible. However, only four feasible and physiologically relevant combinations of PGE2, IL‐6, and CRP have been demonstrated in this proof‐of‐concept study. These four states correspond to the output Boolean or digital logical states for disease endotype and severity analysis. The truth table corresponding to the biomarker combinations in the input sample, the associated Boolean state, and the example suggesting the perceived outcome have been depicted in Figure *6*.

### 4.5 Experimental design of human subject and ELISA studies

The human subject samples were collected as part of approved Institutional Review Board protocols (STU 082010‐016, MR 17‐120). Patient samples were collected during their visit to UTSW Medical Center and written consent was obtained before sample collection and participation in the study. As a proof of concept, three postmenopausal female subjects with active, symptomatic UTIs as diagnosed by clinical urine culture were studied. ELISA kit for PGE2 was purchased from Enzo Life Sciences Inc. (Farmingdale, NY, USA), IL‐6 was purchased from Abcam (Cambridge, MA, USA), and that for CRP was purchased from Sigma Aldrich (St. Louis, MO, USA). *Clean*‐catch urine collected from these patients was aliquoted and stored at −20 C and was retrieved later for analysis. Postanalysis was done by using the calibration dose–response curves for both methods described in the previous sections. Graphics were drawn using Biorender.com *an*d Inkscape v1.

### 4.6 Machine learning studies

Our models were built on Google's CoLab platform, which relies on a quad‐core Intel Xeon processor running at 2.00 GHz per core, 24 gigabytes of ram, and a Tesla V100‐SXM2‐16GB GPU. The model created was a “double random forest” model, which had two stages. The first stage was used to down‐select important features and using only these features the model was developed in the second stage. This was done to create an optimized base model with reduced computational costs that would feed the blended model. Sklearn library in Python was used, and bootstrapping was done randomly with replacement. The optimal number of trees for the simple random forest model, that is, RF1, and that for the optimized RF, that is, RF2 were chosen based on the number of trees that yield the maximum accuracy. The predictions of decision trees were bagged to get the final output class. The choice of random forest is dictated by the fact that this method is a power ensemble method (solves for the bias and high variance issues encountered in decision trees), works very well even with missing data, and is not affected drastically when new data are added. The capability to delineate the most important features based on random forest convergence was yet another important reason to choose it as the base learner model.

### 4.7 Statistical analyses

The data in Figure 5a–c are represented as a Box and Whiskers plot (with min and max points as whiskers) with *n* = 9 (*n* = 3 intersensor and *n* = 3 intrasensor replicates). Similarly, Figure 5d–f has been represented as a Box and Whiskers plot (with min and max points as whiskers). The significance test was carried out using Student's t‐test and one‐way ANOVA with an α of 0.05. All the statistical analyses were performed using Graph Pad Prism version 9.1.2 (GraphPad Software Inc., La Jolla, CA, USA).

## AUTHOR CONTRIBUTIONS

**Antra Ganguly:** Conceptualization (equal); data curation (equal); formal analysis (equal); investigation (equal); methodology (equal); software (equal); validation (equal); visualization (equal); writing – original draft (equal); writing – review and editing (equal). **Tahmineh Ebrahimzadeh:** Data curation (supporting); methodology (supporting); validation (equal); writing – original draft (equal); writing – review and editing (equal). **Jessica Komarovsky:** Data curation (supporting). **Philippe Zimmern:** Conceptualization (equal); investigation (equal); project administration (equal); supervision (equal); writing – original draft (equal); writing – review and editing (equal). **Nicole De Nisco:** Conceptualization (equal); formal analysis (equal); funding acquisition (lead); investigation (equal); project administration (equal); resources (equal); writing – review and editing (equal). **Shalini Prasad:** Conceptualization (equal); formal analysis (equal); investigation (equal); methodology (equal); project administration (equal); resources (equal); supervision (equal); visualization (equal); writing – original draft (equal); writing – review and editing (equal).

## CONFLICT OF INTEREST

Dr. Shalini Prasad has a significant interest in EnLiSense LLC, a company that may have a commercial interest in the results of this research and technology. The potential individual conflict of interest has been reviewed and managed by the University of Texas at Dallas, and it played no role in the study design; the collection, analysis, and interpretation of data; the writing of the article; or the decision to submit the article for publication. The funders had no role in the design of the study; in the collection, analyses, or interpretation of data; in the writing of the manuscript, or in the decision to publish the results.

### PEER REVIEW

The peer review history for this article is available at https://publons.com/publon/10.1002/btm2.10437.

## Supporting information


**Appendix S1:** Supporting Information

## Data Availability

The datasets generated during and/or analyzed during the current study are available from the corresponding author upon reasonable request.
