# Supplementary material for: DigEST: Digital plug‐n‐probe disease Endotyping Sensor Technology
Source: Bioeng Transl Med. 2022 Nov 5;8(2):e10437. doi: 10.1002/btm2.10437 (PMC10013760; doi:10.1002/btm2.10437)
Supplement: Supplementary file 1 — Appendix S1: Supporting Information [file BTM2-8-e10437-s001.pdf]

DigEST: Digital plug-n-probe disease Endotyping Sensor Technology for time-critical disease severity stratification for point-of-care diagnostics

Antra Ganguly,[1] Tahmineh Ebrahimzadeh,[2] Jessica Komarovsky,[2] Philippe E. Zimmern,[3] Nicole J. De Nisco,[2] Shalini Prasad[1*]

*Corresponding author. Email: Shalini.Prasad@utdallas.edu

Supplementary Information

Equivalent circuit modelling of the electrical double layer interface

Fig. S1 below shows the variation in the circuit parameters Rs, Cdl and Rp as a function of the four digital states viz., LLL (State 1), HLL (State 2), HHL (State 3) and HHH (State 4) for (a) PGE2, (b) IL-6 and (c) CRP. For each of the subfigures, the color gradient (light or dark) reflects the variation in the levels (high or low) of the corresponding biomarker expressed in urine. For example, consider (b) which shows the graph for IL-6. Here, for states 1 and 2, the level of IL-6 spiked in the cocktail is low i.e., (LLL and HLL) and hence has been depicted in light red color. On the other hand, for the states 3 and 4, the level of IL-6 spiked in the cocktail is high i.e., (HHL and HHH) and hence has been depicted in dark red color.

This equivalent circuit model analysis was done to evaluate if the individual circuit elements could be tuned to calibrate the sensor for disease endotyping. The relation between Zmod and the circuit elements has been discussed in the supplementary information. From the Fig. S1 (a-c), it is evident that the solution resistance Rs (depicted by squares) is almost constant across the four digital output states. This is favorable as it indicates that EIS is truly mapping the interface and the output impedance is unaffected by the effect of the non-specific molecules and ions in the bulk solution. Next, it was found that for (a) and (b), Cdl (triangle symbol) and Rp (circle symbol) were able to follow the low and high dose concentrations for PGE2 and IL-6 across the four different states. This is because EIS output is very specific to the affinity binding of the target antigen to the specific monoclonal antibody at the double layer interface. However, in figure 1 (c), this effect is not observed, and the individual parameters are unsuitable for disease state endotyping.

Relation between Zmod and the circuit elements(*1*):

1. **Modulus of impedance (Zmod)=**$\sqrt{\mathbf{Zreal^2 + Zimag^2}}$
   Where Zreal is the real component of the complex impedance and Zimag is the imaginary component of the complex impedance.
2. $\mathbf{Zreal = R_s + (R_p/(1+\omega^2 R_p^2 C_{dl}^2)}$
3. $\mathbf{Zimag = \omega R_p^2 C_{dl}/(1+\omega^2 R_p^2 C_{dl}^2)}$

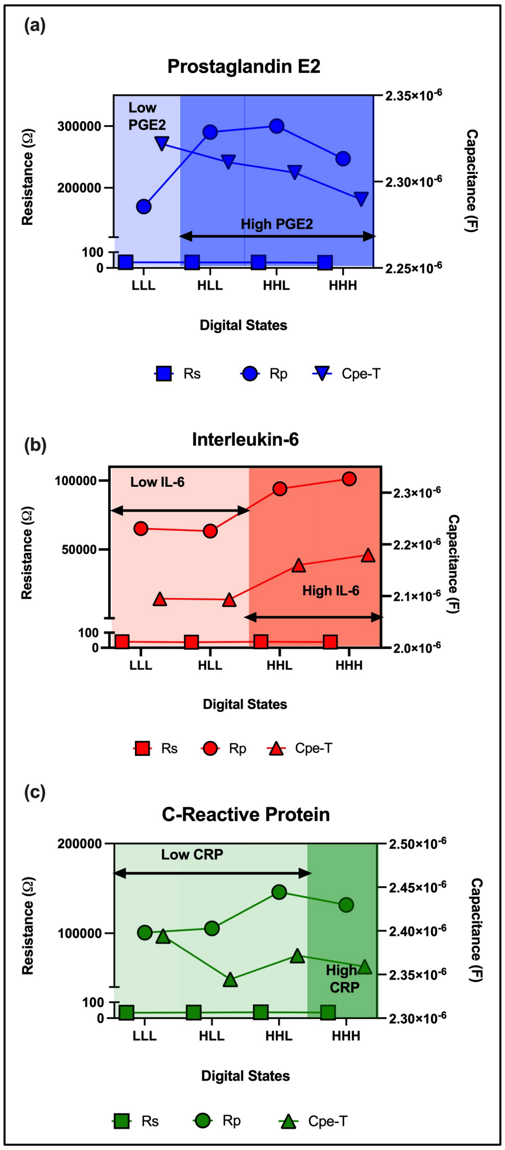

Fig. S1. Equivalent circuit parameters represented for the 4 output digital states for **(a) PGE2, (b) IL-6 and (c) CRP.**

<u>Sensor metrics for PGE2, IL-6 and CRP detection:</u>
The sensor performance was analyzed to evaluate the ability of the sensor to differentiate between specific signal (due the binding of the target biomarker i.e., PGE2, IL-6 or CRP with the corresponding capture probe antibody at the electrical double layer interface) and non-specific bulk species, water and ions present in the urine. The limit of detection (LOD) was defined as the lowest concentration of the biomarker for which the output impedance signal showed significant difference from that due to non-specific blank human urine sample.

The LOD was found to be 1000 pg/mL, 1 pg/mL and 10 pg/mL for PGE2, IL-6 and CRP respectively. As shown in figure S2, statistically significant difference between the non-specific signal and the signal corresponding to the LOD was found for all the 3 biomarkers with with p=

0.0142 for PGE2, p= 0.0040 for IL-6 and p= 0.0005 for CRP using un-paired, Mann Whitney test (α=0.05). Thus, the calibrated sensor shows the specificity to distinguish the inflammatory signal due to UTI from the noise in the urine in the sensor microenvironment.

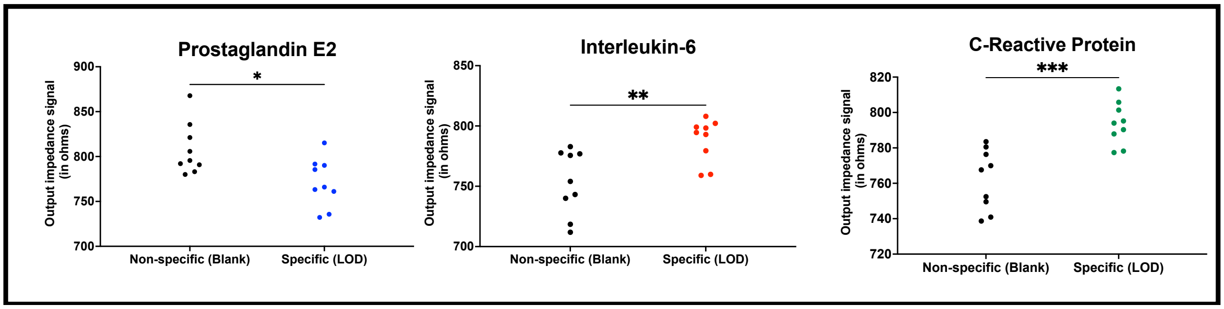

Fig. S2. Comparison of non-specific and specific output impedance signals for **(a) PGE2, (b) IL-6 and (c) CRP.**

Next, to further test the reliability and the repeatability of the sensor response, precision analysis of the sensor response to real pooled human urine samples was done. This was done by studying the coefficient of variation of the output impedance signal.

Coefficient of variation (%CV) = (Standard Deviation/Mean) of the output signal*100

Figure S3. Shows the result of the precision analysis. The coefficient of variation (CV%) was calculated by averaging over all the replicates as a function of the PGE2 levels spiked in urine samples. It was found that the CV% for all the biomarker doses covering the entire dynamic range of the sensor fall well within the acceptable range of 20% set by Clinical laboratory Standards Institute (CLSI) guidelines(*2*, *3*). In this way, it was concluded that the sensor is capable of precise, reliable measurements in real pooled human urine samples using Electrochemical Impedance Spectroscopy with very low variability.

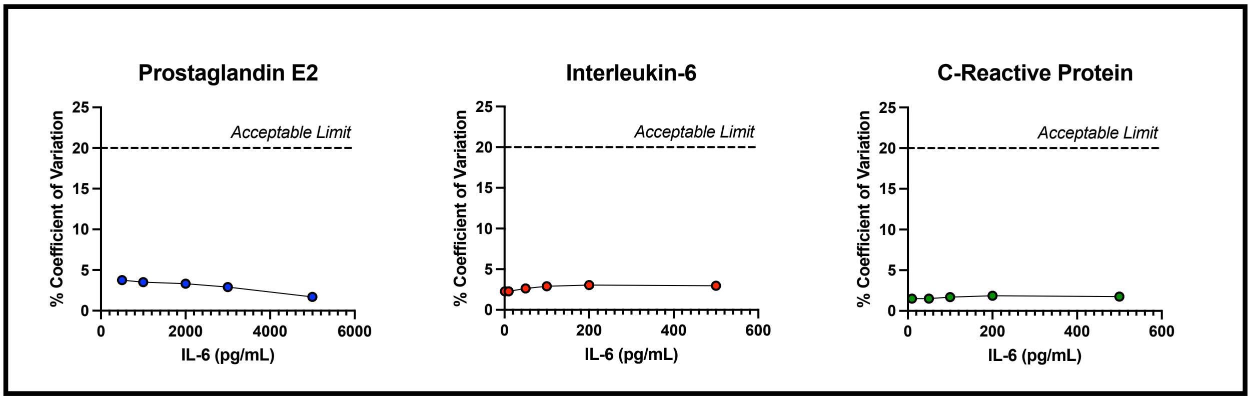

Fig. S3. Reliability and Precision analysis of impedance output for **(a) PGE2, (b) IL-6 and (c) CRP.**

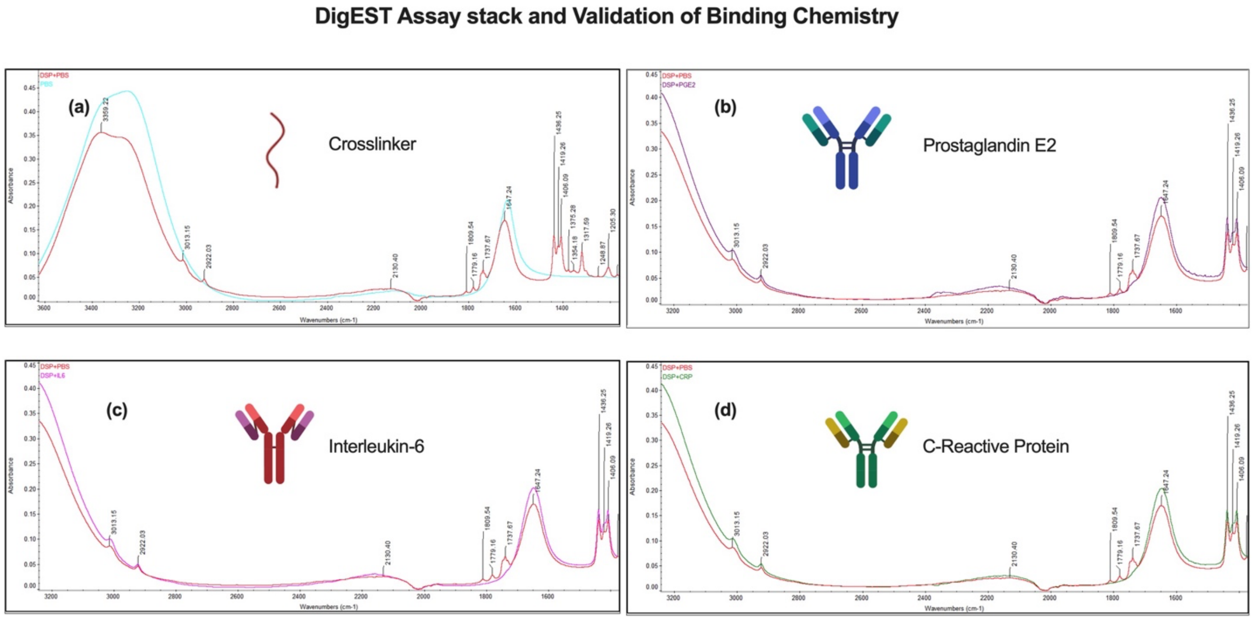

Fig. S4. Attenuated Total Reflectance Fourier Transform Infrared Spectra for **(a) DSP-Cross-linker, (b) PGE2, (c) IL-6 and (d) CRP.**

References:

1. E. P. Randviir, C. E. Banks, Electrochemical impedance spectroscopy: An overview of bioanalytical applications. *Anal. Methods* (2013), , doi:10.1039/c3ay26476a.
2. R. Carey, F. Anderson, H. George, A. Hartmann, V. Janzen, A. Kallner, J. Levine, J. Schiffgens, A. Srinivasan, D. Tholen, User Verification of Performance for Precision and Trueness; Approved Guideline—Second Edition. CLSI document EP15-A2. *CLSI Doc. EP15-A2*. **25** (2006).
3. D. W. Tholen, M. Anders Kallner, J. W. Kennedy Jan S Krouwer, K. Meier, Volume 24 Number 25 Evaluation of Precision Performance of Quantitative Measurement Methods; Approved Guideline-Second Edition (available at www.nccls.org).
